# Prevalence and molecular epidemiology of rotavirus gastroenteritis among children in Nairobi’s urban informal settlements following introduction of the Rotavac® vaccine

**DOI:** 10.1186/s41182-026-00917-7

**Published:** 2026-02-06

**Authors:** Winfred Mbithi, Ernest A. Wandera, Anthony K. Nyamache, Daniel Hungerford, Amos Njuguna, Michael Mugo, Aoko Johnpaul Ogutha, Christine Kioko, Darius Ideke, Carlene Sang, James Nyangao, Phelgona Otieno, Fredrick Were, Khuzwayo C. Jere, Nigel A. Cunliffe, Samuel Kariuki, Cecilia Mbae

**Affiliations:** 1 Kenyatta University, Nairobi, Kenya; 2 Kenya Medical Research Institute, Nairobi, Kenya; 3 Malawi-Liverpool Wellcome Programme, Blantyre, Malawi; 4 University of Liverpool, Liverpool, UK; 5 Drugs for Neglected Diseases Initiative Eastern Africa, Nairobi, Kenya

**Keywords:** Rotavirus gastroenteritis, Vaccine effectiveness, Rotavac®, Strain distribution, Urban informal settlement, Kenya

## Abstract

**Background:**

Rotavirus is the leading cause of acute gastroenteritis among children under five years of age globally. In Kenya, rotavirus vaccination was introduced in 2014 using Rotarix® (G1P[8]), with a subsequent national transition to Rotavac® (G9P[11]) vaccine, in 2023. Evidence on post-introduction rotavirus disease burden, strain diversity, and Rotavac® vaccine effectiveness in Kenya remains limited. This study assessed the burden of rotavirus gastroenteritis and vaccine effectiveness of the rotavirus vaccine among children under five years of age in Nairobi’s urban slums, after the rollout of the Rotavac® vaccine.

**Methods:**

In this cross-sectional surveillance study, 353 stool samples were collected from children under five years of age presenting with acute gastroenteritis at selected health facilities in Mukuru informal settlement, Nairobi, between October 2023 and November 2024. The samples were analyzed using TaqMan array card PCR and multiplexed semi-nested RT-PCR. Vaccine effectiveness for overall rotavirus vaccination and Rotavac® specifically was estimated using a post-hoc test-negative case–control analysis.

**Results:**

Rotavirus was detected in 19.5% (69/353; 95% CI 15.5–24.1%) of the samples. The highest detection occurred among children aged 12–23 months at 24.4% (30/123; 95% CI 17.1–33.0%), with significant differences across age groups (p = 0.023). Rotavirus prevalence was significantly lower among vaccinated children 18.4% (60/327; 95% CI 14.3–23.0%), compared with unvaccinated children 34.6% (9/26; 95% CI 17.2–55.7%) (p = 0.044). The predominant strain was G2P[4] (29.0%; 20/69), which also dominated among the vaccinated children (31.7%; 19/60), while G12P[6] was most frequent among unvaccinated children (33.3%; 3/9). Newly detected strains included G2P[8] and G12P[8], and two equine-like strains (G3P[8]eG3 and G2G3P[4]P[8]eG3). Short electropherotypes predominated. First-dose vaccine coverage was 92.6% (327/353) while the last-dose coverage was 76.2% (269/353). Estimated Rotavac® vaccine effectiveness was 74.1% (95% CI 16.3–92.0%), and overall rotavirus vaccine effectiveness was 57.6% (95% CI 18.1–99.8%).

**Conclusion:**

Rotavirus remains a significant cause of gastroenteritis among children in Nairobi’s urban informal settlements. The circulation of diverse and emerging strains underscores the need for continued molecular surveillance to monitor vaccine performance and guide future immunization strategies.

## Background

Rotavirus is the predominant cause of acute infectious diarrhea among children under five years of age globally [1]. In low- and middle-income countries (LMICs), most children experience their first rotavirus diarrhea episode within the first year of life, in contrast to high-income countries (HICs) where primary infections occur later [1]. Of the estimated 443,833 global deaths from diarrhea in children under five years of age in 2021, 108,322 were attributed to rotavirus diarrhea [2]. The majority of deaths from severe acute rotavirus diarrhea occur in Africa and Asia [3]. In Kenya, rotavirus infection has been estimated to cause over 3000 deaths annually among children under five years of age [4].

To mitigate this burden, four oral live-attenuated rotavirus vaccines; Rotarix® (RV1), RotaTeq, Rotavac® and ROTASIIL, have been prequalified by the World Health Organization (WHO) [5]. Kenya introduced the rotavirus vaccine, Rotarix®, into the routine immunization program in July 2014 [6]. Following its introduction, substantial reductions in rotavirus prevalence were observed across all age groups, with the highest decline occurring among vaccine-eligible infants under one year of age [7].

In January 2023, Kenya transitioned from Rotarix® to Rotavac®, a monovalent, live-attenuated, oral vaccine (116E), administered as a three-dose regimen at 6, 10 and 14 weeks of age. This switch was primarily driven by a global shortage of the Rotarix®. However, evidence on the epidemiological impact and effectiveness of Rotavac® outside India, where the vaccine was developed and evaluated, remains limited. Consequently, there is a critical need to assess the disease burden, strain distribution, and vaccine effectiveness in Kenyan children following the national vaccine transition.

Rotavirus genome comprises 11 segments of double-stranded RNA (dsRNA), which encode 12 proteins (VP1-4, VP6, VP7, NSP1-6). The outer capsid proteins, VP7 and VP4, elicit neutralizing antibodies and define the G (glycoprotein) and P (protease-sensitive) genotypes, respectively [8]. Characterization of these gene segments forms the basis of the dual classification system widely used in epidemiological studies of the human *Rotavirus alphagastroenteritidis (R. alphagastroenteritidis)* [9]. Globally, the most commonly circulating genotypes include G1P[8], G2P[4], G3P[8], G4P[8], G9P[8] and G12P[8] [10, 11]. However, their distribution varies by region and over time. In Kenya, prior to vaccine introduction, G1P[8] was the predominant circulating genotype, followed by G8P[4], G9P[8], G2P[4] and G3P[6]. During the early post- Rotarix® period, G1P[8] remained dominant, but was later replaced by G3P[8] in the late post-vaccine period [12, 13]. In addition to genotyping, electropherotype analysis enables classification of strains into long or short RNA migration profiles, which helps distinguish strains with similar genotypes but different RNA migration profiles. This may reflect genetic variation not captured by VP7 and VP4 genotyping alone [9]. It also identifies the two distinct genotype constellations of *R. alphagastroenteritidis* strains, DS-1-like and Wa-like.

Despite the long-standing use of rotavirus vaccines in Kenya, data on the rotavirus burden and strain diversity following the recent transition to Rotavac® vaccine remain scarce. Given the change from a G1P[8] to a G9P[11]-based vaccine, it is important to evaluate ongoing transmission dynamics, monitor potential shifts in circulating genotypes, and assess vaccine effectiveness under real-world conditions. In this epidemiological surveillance study, we sought to determine the prevalence, vaccine effectiveness, and rotavirus strains most frequently associated with childhood diarrhea in Nairobi’s urban slums, following the introduction of Rotavac® vaccine into Kenya’s national immunization program.

## Methods

### Sample size determination

The sample size was calculated based on the reported prevalence of rotavirus infection among children under five years of age in Nairobi, from a previous study [14]. The minimum required sample size was determined using the formula: n = z^2^p(1-p)/d^2^; where n = represents the minimal sample size, z is the standard normal deviation equivalent to a 95% confidence interval (1.96), p is the estimated prevalence of rotavirus gastroenteritis in children under five years (24%), and d is the degree of precision (0.05). Based on this calculation, a minimum of 280 samples was required. Participant recruitment continued until the predetermined study period ended, resulting in the enrolment of 353 participants.

### Study design and setting

This observational hospital-based surveillance study was conducted in the Mukuru urban informal settlement, which is located 15 km East of Nairobi city, Kenya. It is one of the largest slums in the country with an estimated population of approximately 800,000 residents [15]. The settlement is characterized by overcrowding, poor sanitation, limited access to clean water, and open drainage systems, conditions that facilitate transmission of enteric pathogens including rotavirus. Participant recruitment and sample collection were carried out at four outpatient health facilities serving the Mukuru community: Medical Missionaries of Mary, Mukuru Health Center, Lady of Nazareth Hospital and Reuben Hospital.

### Study population and sample collection

From October 2023 to November 2024, fecal samples were collected from children under five years of age presenting with acute diarrhea, defined as the passage of three or more loose stools within a 24-h period, with or without vomiting or fever. Children presenting with bloody diarrhea or diarrhea lasting more than 7 days were excluded from the study. A purposive sampling method was used, whereby participants WHO met the inclusion criteria were recruited into the study after written informed consent was obtained from their parent or guardian. A standardized pathological investigation form developed in accordance with the WHO guidelines [16] was administered to collect demographic information, clinical characteristics, and card-confirmed rotavirus immunization data, thereby minimizing recall bias. Whole stool samples and rectal swabs were collected from the participants into well-labelled cryovials and stored at − 80 °C. Study data were captured electronically using Epicollect5 [17].

### Detection and Genotyping of Rotavirus

Total nucleic acid (TNA) was extracted from the fecal samples using viral DNA Mini Kit (QIAGEN), according to the manufacturer’s instructions. Briefly, 0.18–0.22 g of stool was subjected to lysis under highly denaturing conditions to inactivate RNases, allowing the extraction of intact TNA containing rotavirus RNA. The lysate was centrifuged at 12,700 rpm to allow binding of the viral RNA onto the QIAamp membrane and subsequently washed using buffers AW1 and AW2 to remove contaminants. Purified TNA was eluted with buffer ATE which prevents microbial growth and RNase-mediated degradation [18]. Rotavirus detection and genotyping were performed using TaqMan array card PCR (TACPCR) according to the manufacturer’s instructions. This method utilizes nucleic acid probes complementary to internal regions of the target RNA [19]. The PCR reactions were run as uniplex assays, with each reaction targeting a specific rotavirus genotype. A threshold cycle (Ct) value was defined as the PCR cycle at which the fluorescence of the reaction exceeded a software-defined threshold. Samples with Ct values between 35 and 40 were considered negative [20].

### Reverse Transcription Polymerase Chain Reaction (RT-PCR)

Multiplexed semi-nested RT-PCR was performed on rotavirus-positive samples whose G and P genotypes could not be identified using TACPCR. Purified rotavirus dsRNA was extracted using QIAamp viral RNA kit according to the manufacturer’s protocol, followed by reverse transcription to generate complementary DNA (cDNA). Multiplex PCR was conducted on the cDNA, followed by nested PCR of the amplified VP4 and VP7 genes. Genotyping of VP4 was performed using primers specific for P[4], P[6], and P[8] while VP7 genotypes were identified using primers specific for G1, G2, G3, G4, G8, G9, and G12 as described previously [21, 22].

### Polyacrylamide Gel Electrophoresis (PAGE)

Polyacrylamide Gel Electrophoresis (PAGE) was used to determine the rotavirus electropherotypes, based on RNA migration patterns of the rotavirus genome. RNA extracted from rotavirus-positive samples, alongside long and short-profile reference strains, was electrophoresed on a 30% polyacrylamide gel. The samples were loaded into 4.2 mm-wide wells and allowed to migrate at 15 mA and 55 V for 17 h. Following electrophoresis, RNA segments were visualized using silver nitrate staining.

### Statistical analysis

Collected data were cleaned and organized using Microsoft Excel 2021 and analyzed using Stata statistical software (version 15.0, Stata Corporation, College Station, TX). Descriptive statistics were used to calculate the overall prevalence of rotavirus infection. Associations between rotavirus positivity and demographic variables were assessed using chi-square tests. The results were summarized and presented using tables and graphical displays. A post-hoc test-negative case–control analysis was conducted to estimate vaccine effectiveness. Crude odds ratios (ORs) were calculated, and vaccine effectiveness was estimated as (1-OR) $\times$ 100% [23]. A 2 × 2 contingency table was constructed to evaluate the association between rotavirus vaccination status and laboratory confirmed-rotavirus infection, with rotavirus-positive children classified as cases and rotavirus-negative children as controls. Severity of the gastroenteritis was assessed using the 20-point Vesikari Clinical Severity Scoring System [24].

## Results

### Demographic characteristics of the study population

A total of 353 children under five years of age were enrolled in the study, and stool samples were successfully collected from all participants. Of these samples, 47.6% (168/353) were whole stool specimen, while 52.4% (185/353) were rectal swabs. Male participants accounted for 52.1% (184/353) of the study population, and females comprised 47.9% (169/353). The mean age of the participants was 19.0 months **(**95% CI 17.0–20.0%). The largest proportion of participants were aged ≤ 11 months (35.4%; 125/353) followed by those aged 12–23 months (34.8%; 123/353) and the least were between 24–59 months (29.7%; 105/353).

### Prevalence of rotavirus infection

Overall, rotavirus was detected in 19.5% (69/353) of the samples (95% confidence interval (CI): 15.5–24.1%). Rotavirus detection varied significantly by age group (p = 0.023), with the highest prevalence observed among children aged 12–23 months at 24.4% (30/123; 95% CI 17.1–33.5%). Children aged ≤ 11 months had a prevalence of 20.8% (26/125; 95% CI 14.1–29.0%) while those aged 24–59 months had the lowest prevalence at 12.4% (13/105; 95% CI 6.8–20.2%). Rotavirus was detected in 18.5% (34/184; 95% CI 13.1–24.9%) of male participants and 20.7% (35/169; 95% CI 14.9–27.6%) of female participants; this difference was not statistically significant (p = 0.597). Among the vaccinated children, rotavirus was detected in 18.3% (60/327; 95% CI 14.3–23.0%), compared with 34.6% (9/26; 95% CI17.2–55.7%) among unvaccinated children, indicating a statistically significant association between vaccination status and rotavirus infection (p = 0.044). These findings suggest that rotavirus vaccination significantly reduced the risk of rotavirus-associated diarrhea (Table 1).

**Table 1:** Comparison between age, sex and immunization data of participants with rotavirus gastroenteritis results by Chi-Square test (n = 353)

	Rotavirus positive cases n = 69	Estimated prevalence % (95% CI)	p-value
Overall prevalence	69/353	19.5 (15.5–24.1)	
Sex			0.597
Male	34/184	18.5 (13.1–24.9)	
Female	35/169	20.7 (14.9–27.6)	
Age (months)			**0.023**
≤ 11	26/125	20.8 (14.1–29.0)	
≤ 2	2/7	28.6 (3.7–71.0)	
3–5	2/34	5.9 (0.7–19.7)	
6–8	12/39	30.8 (17.0–47.6)	
9–11	10/45	22.2 (11.2–37.1)	
12–23	30/123	24.4 (17.1–33.0)	
24–59	13/105	12.4 (6.8–20.2)	
Immunization status			**0.044**
Yes	60/327	18.4 (14.3–23.0)	
No	9/26	34.6 (17.2–55.7)	
Vaccine type			**0.047**
Rotarix®	10/84	11.9 (5.9–20.8)	
Rotavac®	44/222	19.8 (14.8–25.8)	
Mixed	6/21	28.6 (11.3–52.2)	
Unvaccinated	9/26	34.6 (17.2–55.7)	

Rotavirus cases were identified throughout the surveillance period, with the highest proportion occurring between March and May 2024, accounting for 44.9% (31/69) of all detected cases. The lowest number of cases were observed between July and September at 7.2% (5/69), corresponding to the cooler and drier season in Kenya.

### Clinical severity of rotavirus-specific gastroenteritis

Most children with laboratory-confirmed rotavirus infection experienced diarrhea lasting between one and four days (82.6%). The most common frequency of diarrheal episodes was four to five episodes per day (43.5%). Vomiting was frequently reported, with 46.4% of children experiencing two to four episodes per day. The majority of affected children (91.3%) required rehydration only, while 8.7% required hospitalization. Overall, severe gastroenteritis was observed in 46.4% of the rotavirus-positive cases, moderate disease in 37.7%, and mild disease in 15.9%. One rotavirus-associated death was recorded during the study period (Table 2).

**Table 2:** Rotavirus disease severity of rotavirus-associated acute gastroenteritis in Mukuru informal settlement, Nairobi Kenya, 2023–2024 (n = 69)

Clinical parameter	n (%)
Frequency of diarrhea per day	
1–3	10 (14.5)
4–5	30 (43.5)
≥ 6	29 (42.0)
Duration of diarrhea (days)	
1–4	57 (82.6)
5	8 (11.6)
≥ 6	4 (5.8)
Frequency of vomiting per day	
0	11 (15.9)
1	6 (8.7)
2–4	32 (46.4)
≥ 5	20 (29.0)
Duration of vomiting (days)	
0	11(15.9)
1	2 (2.9)
2	24 (34.8)
≥ 3	32 (46.4)
Temperature (°C)	
≤ 37	40 (58.0)
37.1–38.4	22 (31.9)
38.5–38.9	3 (4.3)
≥ 39.0	4 (5.8)
Dehydration	
None	48 (69.6)
1–5%	15 (21.7)
≥ 6%	6 (8.7)
Treatment	
Rehydration	63 (91.3)
Hospitalization	6 (8.7)
Severity category	
Mild	11 (15.9)
Moderate	26 (37.7)
Severe	32 (46.4)

### Rotavirus vaccine effectiveness (VE)

The overall effectiveness of rotavirus vaccine in preventing rotavirus infection was estimated at 57.6% (95% CI 18.1–99.8%) among children under five years. This result indicates that vaccinated children were substantially less likely to develop rotavirus infection than unvaccinated children. The estimated crude Rotavac® vaccine effectiveness was 74.1% (95% CI 16.3–92.0%). The wide confidence intervals observed reflect the small number of unvaccinated children in this study (Table 3).

**Table 3:** Association between overall vaccination status, Rotavac® vaccination and rotavirus infection among children in Mukuru informal settlement

	Cases	Controls	Total
Overall vaccine effectiveness			
Vaccinated	60	267	327
Unvaccinated	9	17	26
Total	69	284	353
Rotavac® vaccine effectiveness			
Vaccinated	50	193	243
Unvaccinated	6	6	12
Total	56	199	255

### Rotavirus G and P Genotype Distribution

All rotavirus positive samples (n = 69) were initially genotyped using TACPCR. Of these, 17.4% (12/69) could not be genotyped using this method and were further analyzed using RT-PCR. Rotavirus G genotypes were successfully identified in 82.6% (57/69) of the positive samples, while 17.4% (12/69) could not be G-genotyped. Genotype G2 was the most predominant, accounting for 52.6% (30/57) of the samples, followed by G3 at 31.6% (18/57) and G12 at 14.0% (8/57). Mixed G2/G3 genotypes were detected in 1.8% (1/57) of samples.

Rotavirus P genotypes were identified in 75.4% (52/69) of the samples. P[4] was the most common P genotype detected in 40.4% (21/52) of cases, followed by P[8], P[6] and P[4]P[8], at 34.6% (18/52), 15.4% (8/52), and 9.6% (5/52), respectively. P genotypes could not be determined in 24.6% (17/69) of the rotavirus-positive samples.

**Fig. 1:**
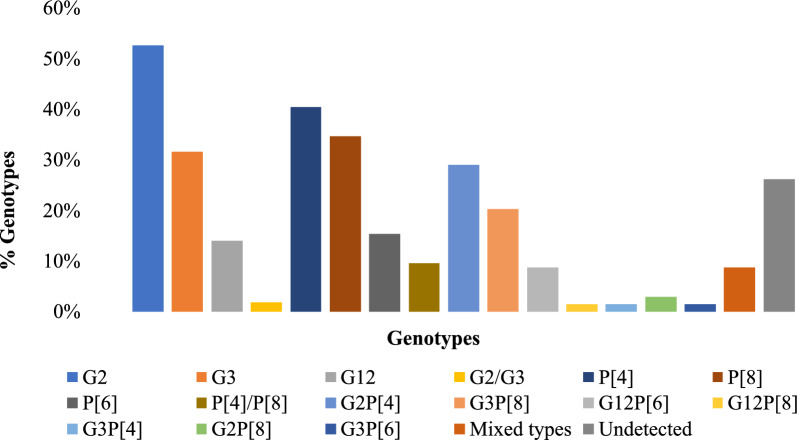
Distribution of rotavirus genotypes detected in stool samples showing the proportion of G-types (G2, G3, G12 and G2/G3), P-types (P[4], P[8], P[6]), P[4]/P[8], combined G-P genotypes (G2P[4], G3P[8], G3P[4], G12P[6], G12P[8], G3P[4], G2P[8], G3P[6]), mixed genotype infections and samples with no detectable genotype (n = 69)

**Fig. 2:**
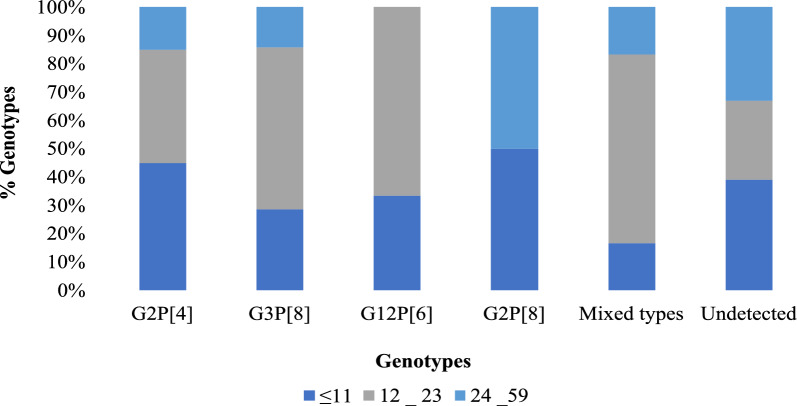
Percentage distribution of rotavirus genotypes (Y-axis) by age group (X-axis) in Mukuru slums post-vaccine introduction. Age groups: ≤ 11, 12–23 and 24–59 months (n = 69)

**Fig. 3:**
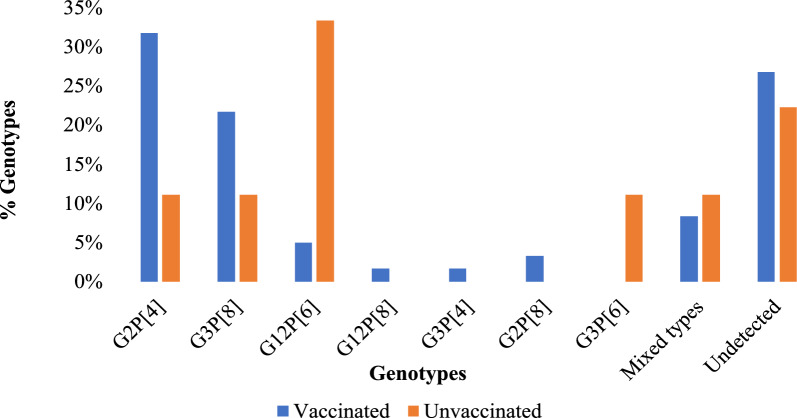
distribution of rotavirus genotypes by vaccination status in the Mukuru slums (n = 69). Percentages of each genotype are shown on the Y-axis, with vaccination status and genotypes on the X-axis. Data include vaccinated (n = 60) and unvaccinated (n = 9) children

The most dominant G–P combination was G2P[4], accounting for 29.0% (20/69) of all the rotavirus-positive samples. This was followed by G3P[8] (20.3%; 14/69), G12P[6] (8.7%; 6/69) and G2P[8] (2.9%; 2/69). Less frequent G–P combinations included G12P[8], G3P[4], G3P[6] each detected in 1.4% (1/69) of samples. Three mixed-genotype infections, two of which were equine-like (G3P[8]eG3 and G2G3P[4]P[8]eG3) and one G2P[4]P[8], were detected in 8.7% (6/69) of samples. Overall, 26.1% (18/69) of the positive samples could not be typed for both P and G types (Fig. 1). Mixed genotypes and G3P[8] were most frequently detected among children aged 12–23 months, accounting for 66.7% (4/6) and 57.1% (8/14) respectively (Fig. 2). Among vaccinated children, G2P[4] was the predominant strain at 31.7% (19/60), whereas G12P[6] was most frequently detected among unvaccinated children at 33.3% (3/9) (Fig. 3).

### Electropherotypes

**Fig. 4:**
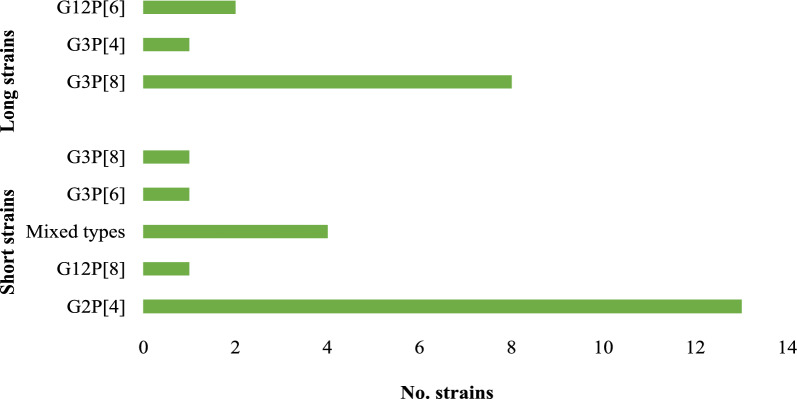
Distribution of rotavirus electropherotypes and genotypes among children in Mukuru informal settlement after rotavirus vaccine introduction. Electropherotypes, categorized as long or short strains based on the migration patterns of the 11 viral RNA segments on polyacrylamide gel electrophoresis, are shown on the Y-axis (n = 31)

Of the 69 rotavirus-positive samples, 56 had a sufficient material for electropherotype analysis using PAGE. Short strains predominated across all age groups, accounting for 39.3% (22/56) of samples while long strains were observed in 19.6% (11/56). No visual bands were obtained in 41.1% (23/56) of samples. DS-1-like G2P[4] strains were predominantly detected among short electropherotypes accounting for 59.1% (13/22) of samples, while Wa-like G3P[8] was mostly detected in long electropherotypes 72.7% (8/11). Notably, one Wa-like strain (G3P[8]) exhibited DS-1-like characteristics, suggesting possible reassortment. Among the short strains, 9.1% (2/22) of samples could not be assigned G and P genotypes (Fig. 4).

## Discussion

This study provides updated evidence on the burden, clinical severity, vaccine effectiveness, and molecular epidemiology of rotavirus infection among children under five years of age in the Mukuru informal settlement, Nairobi. *R. alphagastroenteritidis* was detected in 19.5% of children presenting with acute gastroenteritis. This is slightly lower than the 24% prevalence reported in the same setting, prior to rotavirus vaccine introduction in Kenya [14]. These findings are consistent with observed reductions in rotavirus-specific acute gastroenteritis following vaccine implementation in Kenya and other LMICs. In central Kenya, rotavirus-specific acute gastroenteritis (AGE) declined from 27.5% in the pre-vaccine period (2009–2014) to 13.8% in the early post-vaccine period (2014–2016), and further to 12.0% in the late post-vaccine period (2019–2020) [12]. Notably, this study represents, to our knowledge, the first post-introduction evaluation following Kenya’s transition from RV1 to Rotavac® vaccine in 2023.

The age-specific distribution of rotavirus infection observed in this study aligns with established post-vaccine epidemiological patterns, with the highest prevalence occurring among children aged 12–23 months. Similar age shifts have been reported in Kenya, Malawi, China and India, where rotavirus infections are increasingly detected in older children compared to vaccine-eligible infants after vaccine introduction [25–28]. This change likely reflects strong vaccine-derived protection during the first year of life, followed by waning immunity beyond infancy. Several studies from LMICs have documented declining vaccine effectiveness after the first year of life, supporting this interpretation [29, 30]. Additionally, the emergence of novel or heterotypic strains under vaccine-driven selection pressure may further influence age-related susceptibility patterns [31].

The overall vaccine effectiveness of approximately 58% is comparable to estimates reported for Rotarix® in Kenya and similar LMIC settings [32]. Crude vaccine effectiveness for Rotavac® was estimated at approximately 74%, providing encouraging evidence of protection following the national vaccine switch. Reduced vaccine effectiveness in this setting may be partly attributable to environmental enteric dysfunction (EED), which is highly prevalent in resource-limited settings. It has been shown to impair oral vaccine performance through chronic intestinal inflammation and reduced nutrient absorption [33]. Despite lower vaccine effectiveness in African settings, evidence of protection is reassuring in high-burden populations, such as Mukuru slums, where vaccination yields substantial public health impact.

Clinical severity findings indicate that nearly half of rotavirus-positive cases were classified as severe based on Vesikari scores. This proportion is higher than that reported in a post-vaccine hospital-based study at Kenyatta National Hospital, Kenya, which documented severe disease in 28.3% of cases [25]. Differences in recruitment settings, circulating strains timing of the studies relative to rotavirus vaccine introduction may explain this variation. Although most children required only rehydration therapy, the occurrence of hospitalizations and a documented rotavirus-associated death highlight the continued risk of severe disease in vulnerable populations.

Rotavirus cases were recorded throughout the year, with a notable peak between March and May, coinciding with the rainy season in Kenya. This seasonal pattern differs from previous studies in Kenya, Mali, and The Gambia, which reported peaks during the drier seasons [34, 35]. These findings suggest potential shifts in rotavirus seasonality and underscore the need for continued surveillance to better characterize temporal transmission patterns in this setting.

The molecular epidemiology findings revealed a predominance of G2P[4], followed by G3P[8], indicating a marked shift in rotavirus genotype circulation following the introduction of the rotavirus vaccine in Kenya’s immunization program. Prior to vaccine rollout, a study conducted in this setting showed G1P[8] as the predominant strain. The rise of G2P[4], a heterotypic strain relative to the G1P[8]-based Rotarix®, mirrors post-vaccine trends reported in Central Kenya, Kilifi, and Western Kenya, suggesting possible vaccine-induced pressure [12, 36, 37]. The increasing detection of G12P[6], G3P[8], and G2P[8], previously uncommon genotypes, reflects expanding viral diversity and is consistent with observations from Brazil, India and Malawi following RV1 introduction [27, 38, 39].

Globally, the shift toward non-G1 and heterotypic genotypes has been widely documented and has prompted discussions on strain adaptation, cross-protection and long-term vaccine impact. These findings from Mukuru, especially in the context of Kenya’s recent transition to Rotavac®, a G9P[11]-based vaccine, underscore the importance of routine surveillance to monitor whether the trend toward heterotypic and reassortant strains persists in the population.

Electropherotype analysis demonstrated a predominance of short RNA profiles, particularly among G2P[4] strains, consistent with their DS-1-like genomic constellation. They accounted for most infections across all age groups, which contrasts the pre-vaccine era, during which the long strain predominated while the short electropherotype was least detected [14]. The detection of a G3P[8] strain exhibiting a short profile, an atypical finding, suggests reassortment events that may alter RNA migration patterns. These findings highlight the need for advanced molecular approaches, such as whole-genome sequencing, to further characterize the circulating strains.

This study was conducted approximately nine months after the introduction of the Rotavac® vaccine. Vaccine coverage was high for the first dose (92.6%), indicating good access to the vaccine. This is likely supported by free immunization services and ongoing health promotion activities in the community. However, the low coverage of the last scheduled vaccine dose demonstrates a notable dropout rate of 17.7%, indicating difficulties in retaining children within the immunization schedule. Partial vaccination may have contributed to the observed vaccine effectiveness in this study. Evidence indicates that while partial dosing provides some protection against RVGE, this protection is weaker and less durable than that achieved with complete vaccination [40]. Achieving and sustaining high coverage across all recommended doses is essential for maximizing community level protection (41). The high dropout rate shows poor utilization of the vaccine, which could be attributed to frequent movement of the residents. Moreover, given the economic constraints in this area, caregivers may skip vaccination visits to prioritize earning a livelihood. Strengthening caregiver awareness of vaccine benefits is essential for enhancing vaccine utilization and improving child health.

While the findings provide valuable insights, it is not without limitations. The one-year study duration limits assessment of long-term trends in incidence or genotype distribution. Additionally, the small size of vaccine subgroups reduces statistical power and precision, limiting detailed analyses by age or number of doses. The high first-dose coverage may introduce bias as unvaccinated children likely differ from the general population in ways that affect disease risk, independent of vaccination. Lastly, the hospital-based purposive sampling design limits generalizability to children who did not seek care at the selected facilities. Future longitudinal, multi-site studies incorporating robust clinical metadata and advanced genomic techniques are recommended to build on these findings.

In conclusion, rotavirus remains a significant cause of acute gastroenteritis among young children in Mukuru informal settlement, with substantial disease severity and ongoing circulation of diverse and emerging genotypes despite vaccine introduction. These findings highlight the need for sustained surveillance, improved completion of vaccination schedules, and integration of genomic monitoring to inform vaccine policy. In addition, strengthening complementary interventions, including water, sanitation and hygiene (WASH) infrastructure, will be critical to further reducing the burden of rotavirus disease in high-risk informal settlements in Kenya and similar settings. Future studies should evaluate the effectiveness of a booster dose or the neonatal vaccine in sustaining protection and reducing rotavirus disease burden in Kenya.

## Data Availability

The datasets used and/or analyzed during the current study are available from the corresponding author on reasonable request.
